# Virtual reality-assisted pulmonary rehabilitation enhances early lung function recovery after thoracoscopic surgery in lung cancer patients: a non-concurrent controlled study

**DOI:** 10.3389/fmed.2025.1643688

**Published:** 2025-09-04

**Authors:** Wang Wenjie, Jiang Yifan, Wang Li, Guan Xiaomin, Zhou Linlin, Cheng Yuhan, Wan Sibei, Fang Fang

**Affiliations:** ^1^Shanghai General Hospital, Shanghai Jiao Tong University School of Medicine, Shanghai, China; ^2^School of Nursing, Shanghai Jiao Tong University, Shanghai, China; ^3^School of Medicine, Tongji University, Shanghai, China; ^4^Shanghai Tenth People’s Hospital, Tongji University, Shanghai, China

**Keywords:** lung cancer, virtual reality, health education, pulmonary function training, postoperative rehabilitation

## Abstract

**Objective:**

To investigate the clinical effectiveness of pulmonary rehabilitation health education and respiratory function training based on virtual reality (VR) technology in promoting early postoperative pulmonary function recovery in patients undergoing thoracoscopic surgery for lung cancer.

**Methods:**

A single-center, non-concurrent, pre-post controlled study was conducted. Ninety-five patients who underwent thoracoscopic radical resection of lung cancer between June 2023 and February 2024 were assigned to the control group and received routine postoperative respiratory function education. Another 95 patients admitted between March and December 2024 were assigned to the intervention group and received VR-based health education combined with training guidance. The intervention lasted for 3 days. Outcomes compared between groups included pulmonary function recovery, time to extubation, incidence of postoperative pulmonary complications, satisfaction with health education, and safety of the intervention.

**Results:**

The compliance rate in the intervention group was 94.74%, significantly higher than that in the control group (85.26%) (*p* < 0.05). On postoperative day 3, FVC%, FEV_1_%, MVV, and MVV% were significantly better in the intervention group compared to the control group (*p* < 0.01). The time to extubation was also significantly shorter in the intervention group (*p* < 0.01). There was no significant difference in the incidence of pulmonary complications within 7 days after surgery between the two groups (*p* > 0.05). Patient satisfaction in the intervention group was 91.11%, significantly higher than 72.84% in the control group (*p* < 0.01). Three mild adverse events were reported during the VR intervention, which were resolved after temporary suspension of the session and did not affect the completion of the intervention.

**Conclusion:**

VR-based health education combined with postoperative respiratory function training effectively improves patient compliance, promotes early pulmonary function recovery, shortens extubation time, and enhances patient satisfaction. It is clinically safe and feasible and holds promise for broader application in postoperative nursing care for lung cancer patients.

## 1 Introduction

Lung cancer remains one of the leading malignancies worldwide in terms of both incidence and mortality (1). With the advancement of minimally invasive surgical techniques, video-assisted thoracoscopic surgery (VATS) has become the primary surgical treatment for patients with early-stage lung cancer. Compared with traditional open thoracotomy, VATS offers significant advantages in reducing postoperative pain, promoting pulmonary function recovery, and preventing pulmonary infections. However, studies have shown that in the early postoperative period, some patients still experience reduced pulmonary ventilation and incomplete lung re-expansion due to factors such as lobectomy, thoracic drainage, and restricted mobility, which increase the risk of atelectasis and postoperative pulmonary infections (2).

Pulmonary function exercises, as a non-pharmacological and minimally invasive postoperative rehabilitation approach, can enhance alveolar ventilation, improve diaphragmatic activity, and promote sputum clearance, thereby improving respiratory function and oxygenation efficiency, and accelerating postoperative recovery (3). Although exercise guidance is commonly provided in clinical nursing practice, it is often limited to verbal instructions and printed materials, which are abstract, lack feedback, and offer poor interactivity. Postoperative patients are frequently reluctant to participate due to fatigue, anxiety, or insufficient understanding, resulting in poor exercise compliance and performance, which ultimately affects the effectiveness of pulmonary rehabilitation interventions (4).

In recent years, virtual reality (VR), as an immersive, visual, and interactive digital educational tool, has attracted growing attention in the field of medical and nursing care. Siripongsaporn et al. (5) demonstrated that immersive VR education prior to endoscopy significantly enhanced patients’ understanding and recall of procedural information, outperforming traditional verbal instruction in engagement and information retention.

However, research exploring VR’s role in the postoperative rehabilitation phase, particularly its effects on compliance and quality of pulmonary exercises, remains limited—though recent meta-analyses suggest that VR can significantly enhance lung function and emotional wellbeing (6).

In light of this, the present study was designed and implemented to evaluate a pulmonary rehabilitation education and postoperative respiratory exercise intervention based on virtual reality (VR) technology. The aim was to assess its effects on multiple clinical outcomes among patients undergoing thoracoscopic surgery for lung cancer, including pulmonary function recovery, timing of chest tube removal, pain perception, and patient satisfaction. This study seeks to provide empirical evidence and practical guidance for the broader application of VR technology in postoperative rehabilitation nursing.

## 2 Materials and methods

### 2.1 Study population

This study adopted a single-center, non-concurrent, pre-post controlled design. The control group consisted of hospitalized patients who underwent video-assisted thoracoscopic surgery (VATS) for lung cancer between June 2023 and February 2024 and received conventional postoperative pulmonary rehabilitation education. The intervention group comprised patients with similar characteristics who were admitted between March and December 2024 and received a pulmonary rehabilitation intervention based on virtual reality (VR) technology, including health education and guided respiratory exercises. A total of 95 patients were included in each group. The study protocol was approved by the Ethics Committee of the hospital (Approval No. 2023026), and all enrolled patients provided written informed consent.

### 2.2 Inclusion and exclusion criteria

#### 2.2.1 Inclusion criteria

① Age ≥18 years; underwent elective video-assisted thoracoscopic lobectomy with an intraoperative pathological diagnosis of lung cancer; ② no severe active infection prior to surgery; ③ normal cognitive function, able to understand health education content and cooperate with training procedures; ④ expected postoperative hospital stay of no less than 3 days and consent to participate throughout the entire study period.

#### 2.2.2 Exclusion criteria

① Requirement for prolonged mechanical ventilation after surgery; ② underwent complex surgical procedures such as combined chest wall resection; ③ experienced significant discomfort (e.g., dizziness, nausea) during the initial exposure to VR content; ④ transferred to another department or hospital, or voluntarily withdrew from the study within 3 days after surgery.

### 2.3 Intervention methods

#### 2.3.1 Control group

Patients in the control group received standard postoperative care and bedside pulmonary function exercise education. The intervention was initiated within 24 h after surgery. Upon confirming stable vital signs and clear consciousness, the responsible nurse conducted the initial bedside education, emphasizing the importance of postoperative pulmonary function recovery, the purpose of the exercises, and key points for patient cooperation. Based on the patient’s recovery status, the nurse then guided the patient to perform standardized pulmonary rehabilitation exercises, which mainly included the following three components: ① Pursed-lip breathing training: Patients were guided to assume a semi-recumbent position, inhale slowly through the nose, and exhale slowly through pursed lips as if whistling, maintaining an inhalation-to-exhalation ratio of 1:2, 10 repetitions per set; ② Abdominal (diaphragmatic) breathing training: In a supine or sitting position, patients placed both hands on the abdomen, allowing the abdomen to rise during inhalation and retract during exhalation, encouraging deep, slow, and rhythmic breathing, 10 repetitions per set; ③ Effective coughing training: Patients were instructed to perform “deep inhalation—breath-hold for 2–3 s—segmented coughing” to assist sputum clearance and prevent pulmonary infections and atelectasis.

These exercises were conducted twice daily, scheduled at 10:00 a.m. and 4:00 p.m., each session lasting 15–20 min. The responsible nurse provided on-site guidance and monitoring during each session, observing for signs of fatigue, dyspnea, or discomfort. If significant intolerance occurred, timely evaluation and adjustment were performed. The exercise intervention lasted for three consecutive days, during which the nurse documented daily completion status and the patient’s subjective satisfaction, and recorded implementation details in the nursing notes.

#### 2.3.2 Intervention group

On the basis of routine care provided to the control group, the intervention group received an additional VR-based intervention. Centered on the goal of promoting early postoperative pulmonary function recovery, an immersive educational system was developed that integrated both health education and skill training functionalities. The intervention process consisted of four components: team formation, content development, video production, and system implementation. Through the use of technology, the program enabled visualization of educational content, interactivity in exercise training, and structured assessment of training effectiveness. The total duration of the intervention was 3 days, with specific details as follows:

(1) Formation of the VR pulmonary rehabilitation education team: The project was based in the thoracic surgery ward, where a multidisciplinary intervention team was established. The team comprised: one associate chief physician of thoracic surgery (responsible for protocol review and technical support), four resident physicians (responsible for clinical assessment and care coordination), one head nurse (serving as project coordinator and quality control supervisor), two senior nurses certified in pulmonary rehabilitation (responsible for video demonstration and training guidance), and eight bedside nurses (responsible for on-site implementation and observation recording). All team members received standardized training, covering VR equipment operation, educational protocol procedures, emergency response plans, and standardized nursing documentation.
(2) Development of educational content and program framework: The VR content was structured into two modules: “Health Education” and “Rehabilitation Guidance.” The instructional material was developed with reference to domestic and international postoperative rehabilitation guidelines for lung cancer and evidence-based studies published in the past 5 years. Intervention points were designed in stages to align with the patient’s postoperative recovery trajectory. The health education module focused on the significance of postoperative pulmonary function recovery, risks of complications, the value of respiratory exercises, and key points for patient cooperation. The rehabilitation guidance module combined real-life video footage with animation to demonstrate standardized techniques, including pursed-lip breathing, diaphragmatic (abdominal) breathing, and effective coughing. The system supported synchronous learning, enabling patients to “watch and practice” simultaneously (see Tables 1, 2 for details).

**Table 1:** Educational content of pulmonary rehabilitation using VR.

Topic	Key content	VR presentation format
Significance of postoperative pulmonary function recovery	Optimize lung tissue remodeling: promote structural regeneration of the remaining lung tissue after surgery to maintain functional lung reserve.	3D models are used to visualize lung structures and highlight unaffected regions post-resection. Simultaneously, real alveolar expansion is shown to indicate healthy ventilation. When explaining breathing mechanics, the system demonstrates diaphragm motion and airflow patterns interactively.
	Enhance respiratory regulation: The intervention can restore disrupted neuromuscular control of breathing caused by surgery, strengthen the brain’s control over respiratory muscles, reestablish normal respiratory rhythm and depth, and ensure stable oxygen supply.	Animated internal body perspectives are used to illustrate the respiratory-related neural reflex arc, creating an intuitive and immersive visual effect. The animation clearly demonstrates the signal transmission process between the brain and respiratory muscles during breathing exercises, as if opening a window into the inner workings of the human body. For example, light beams are creatively used to represent neural signals, moving in an orderly fashion along neural pathways. Simultaneously, the respiratory muscles are shown rhythmically contracting and relaxing in response to these signals, vividly portraying the progressive restoration of a stable respiratory control mechanism from initial dysregulation.
	Improve respiratory reserve function: After lung cancer surgery, respiratory reserve is reduced. Training can enhance thoracic compliance and respiratory muscle strength, thereby improving respiratory reserve, helping patients meet increased oxygen demands, and reducing activity limitations.	Contrasting real-life scenarios are used to illustrate functional improvement. Initially, the VR displays a patient experiencing shortness of breath during simple activities (e.g., walking a short distance) in the early postoperative stage. The scene then transitions to the same patient, after undergoing pulmonary rehabilitation, performing the same or even more demanding activities (e.g., walking longer distances, climbing stairs) with ease. This panoramic comparison vividly demonstrates the improvement in activity tolerance resulting from enhanced respiratory reserve function.
Common complications risk	Risk of Pulmonary Infection: Postoperative respiratory defense mechanisms are impaired in lung cancer patients. During early rehabilitation, retained sputum and poor pulmonary function recovery can create a favorable environment for bacterial growth, increasing the risk of pulmonary infection.	VR Scene Simulation: In the VR setting, an animated visualization of the lung’s internal microenvironment is presented. It demonstrates the weakened movement of airway cilia following surgical trauma and simulates sputum accumulation within the respiratory tract. Subsequently, the animation depicts how insufficient pulmonary rehabilitation leads to bacterial colonization and proliferation, resulting in inflammatory responses. Visual cues such as reddening and swelling of lung tissues are used to intuitively illustrate the progression and risk of pulmonary infection.
	Postoperative patients are prone to alveolar collapse and subsequent atelectasis due to pain and respiratory muscle weakness. Inadequate rehabilitation strategies further increase this risk, negatively affecting pulmonary ventilation.	A 3D model is used to demonstrate the overall lung anatomy, with a focus on the alveolar structures. The simulation portrays collapsed alveoli caused by shallow breathing due to postoperative pain and reduced respiratory muscle strength. This leads to localized atelectasis and impaired ventilation, which can be observed from multiple angles. The animation then transitions to depict the gradual re-expansion of alveoli during proper pulmonary rehabilitation—such as deep breathing exercises and incentive spirometry—showing improved alveolar inflation and resolution of atelectasis in real time.
	Risk of Deep Vein Thrombosis(DVT): Postoperative patients often experience restricted mobility and a hypercoagulable state. Prolonged bed rest and limited limb movement during pulmonary rehabilitation can lead to venous stasis in the lower extremities, thereby significantly increasing the risk of deep vein thrombosis.	VR Simulation for DVT Education and Prevention: The VR simulation initially presents the internal structure of the lower limb venous system, illustrating the dynamic state of blood flow. It demonstrates how postoperative physical inactivity causes sluggish and viscous blood flow, which progressively leads to thrombus formation as blood elements adhere to the vascular endothelium. The simulation vividly captures the transition from early-stage microthrombi to larger, more obstructive clots. Subsequently, the scene transitions to depict regular limb exercises—such as leg flexion-extension and turning over—conducted in accordance with pulmonary rehabilitation protocols. This part shows the restoration of normal venous blood flow and the associated reduction in DVT risk. The visualization underscores the critical role of physical activity in preventing deep vein thrombosis and highlights the potential dangers of neglecting this component of postoperative care.
	Risk of Respiratory Failure: If pulmonary function training fails to effectively enhance ventilation and gas exchange capacity, the patient’s lung function may progressively deteriorate, potentially leading to respiratory failure—a life-threatening condition.	Visualization of Pulmonary Function via Advanced Virtual Scenarios: Using advanced virtual reality technologies, a comprehensive and anatomically accurate 3D model of the human respiratory system is constructed to fully illustrate its intricate structure. Simultaneously, key indicators of pulmonary function—such as vital capacity (reflecting ventilatory efficiency) and blood oxygen saturation (representing oxygenation status)—are presented through dynamic visualizations, including real-time numerical displays and data trend curves. The simulation depicts a scenario in which pulmonary rehabilitation proves ineffective, resulting in a gradual decline in pulmonary function parameters. As the patient’s breathing becomes increasingly labored, the simulation progresses to a critical stage characterized by clinical signs of respiratory failure, such as cyanosis of the lips and impaired consciousness.
The value of proper exercise	The Importance of Optimizing Breathing Patterns: Pulmonary rehabilitation facilitates the re-establishment of efficient and physiologically appropriate breathing patterns. It helps correct dysfunctional breathing, enabling smoother, more coordinated respiration, enhancing gas exchange efficiency in the lungs, ensuring adequate oxygen delivery to vital organs, and optimizing overall respiratory function.	VR-Based Visualization of Breathing Pattern Optimization: The VR simulation initially depicts a postoperative lung state characterized by disordered respiration, with uncoordinated alveolar expansion and contraction, resulting in unstable oxygen supply. As the patient begins guided respiratory control training, the simulation dynamically shows a transition toward a more stable and efficient breathing rhythm. Alveolar movements become well-coordinated, facilitating gas exchange at an optimal pace. Real-time visual effects across organ systems indicate a steady and sufficient oxygen supply. The simulation is further enhanced with real-time data displays, including respiratory rate and gas exchange efficiency, clearly illustrating the physiological improvement associated with optimized breathing patterns.
	The Value of Enhancing Thoracic Mobility: Appropriate physical exercises can significantly improve thoracic mobility and flexibility, enhancing chest wall compliance. This improved coordination between the thorax and pulmonary structures facilitates more effective lung expansion and ventilation, thereby indirectly boosting pulmonary function. Additionally, increasing thoracic flexibility can alleviate discomfort caused by chest wall restriction and improve overall physical comfort.	VR-Based Visualization of Thoracic-Pulmonary Coordination: Using VR technology, a three-dimensional, interactive visualization of thoracic-lung interaction is constructed. Initially, the simulation presents a postoperative scenario in which thoracic movement appears rigid and restricted, impairing its coordination with lung expansion during respiration. This misalignment is observable from multiple angles, highlighting the mechanical discordance between the chest wall and lungs. Following targeted exercises—such as thoracic stretching and rotation—the simulation displays a gradual increase in thoracic range of motion. The chest wall becomes more flexible and capable of synchronizing smoothly with respiratory rhythms, enabling efficient and complete lung expansion. Consequently, the lungs benefit from improved ventilatory capacity, supporting better pulmonary performance.
	The Value of Promoting Respiratory Muscle Coordination: Proper respiratory training facilitates the coordinated functioning of key respiratory muscles, including the intercostal muscles and diaphragm. Balanced muscular strength and synchronized movements prevent overuse or underutilization of specific muscle groups, ensuring smoother respiratory mechanics and enhancing overall respiratory efficiency.	VR-Based Visualization of Respiratory Muscle Synergy: Through VR technology, the condition of respiratory muscle coordination is vividly demonstrated. Initially, the simulation presents a scenario where the intercostal muscles and diaphragm operate out of sync—characterized by imbalanced force distribution and uncoordinated movement—resulting in labored and inefficient breathing, which compromises pulmonary ventilation. As the patient undergoes targeted respiratory muscle training, such as endurance exercises, the simulation shows progressive improvement. The intercostal muscles and diaphragm begin to contract and relax in a harmonized manner, with more balanced force output. Breathing becomes smoother and more natural, while ventilation efficiency significantly improves. These improvements are further reinforced by dynamic changes in relevant physiological indicators, highlighting the effectiveness of respiratory muscle coordination training.
Key considerations for patient compliance	Adherence to Medical Guidance and the Principle of Gradual Progression: Patients must follow the rehabilitation plan prescribed by their physicians, avoiding arbitrary changes to exercise intensity or frequency. Pulmonary rehabilitation should proceed progressively, beginning with low-intensity activities and gradually increasing as the patient’s condition improves. Hastening the process may result in physical strain or complications, hindering recovery.	VR-Based Demonstration of Postoperative Rehabilitation Compliance: The VR simulation illustrates the rehabilitation journey of a post-lung cancer surgery patient. Initially, it shows a physician developing a personalized rehabilitation plan based on the patient’s condition, specifying exercise phases, intensity levels, and frequency—e.g., three sessions per day of five-minute breathing exercises during the first week, with gradual increases in subsequent stages. The simulation then contrasts two scenarios: one patient who follows the plan experiences steady physical improvement and stable physiological indicators; another patient, who prematurely engages in high-intensity training, develops adverse symptoms such as tachycardia, shortness of breath, and worsened wound pain. This visual comparison underscores the critical importance of adhering to medical guidance and progressing gradually throughout the rehabilitation process.
	Importance of Proper Breathing Posture: Correct posture is essential for effective respiratory training. For diaphragmatic (abdominal) breathing, the patient should lie in a supine or semi-recumbent position with relaxed muscles. Inhalation should be performed through the nose, causing abdominal expansion, followed by exhalation through the mouth as the abdomen contracts. For pursed-lip breathing, the mouth remains closed during nasal inhalation, followed by slow exhalation through pursed lips, with the duration of exhalation longer than inhalation. Improper posture or technique can significantly reduce the effectiveness of the exercise.	VR-Based Simulation of Proper and Improper Breathing Techniques: A VR indoor rehabilitation scenario is constructed to demonstrate correct execution of diaphragmatic and pursed-lip breathing. For diaphragmatic breathing, the patient is shown lying in bed in a relaxed state, with visible abdominal rise during nasal inhalation and fall during exhalation. Voiceover instructions guide the rhythm and key points. In the pursed-lip breathing sequence, the patient inhales through the nose, then exhales slowly through pursed lips in a whistle-like shape, with onscreen visuals indicating the appropriate inhalation-to-exhalation time ratio. The simulation then contrasts with incorrect postures: for example, lack of visible abdominal movement during diaphragmatic breathing or failure to properly purse the lips and exhale too quickly during pursed-lip breathing. These incorrect techniques are shown to result in labored breathing and reduced therapeutic effect, emphasizing the critical role of proper posture and technique in respiratory rehabilitation.
	Balanced Scheduling of Rest and Exercise: Postoperative patients must maintain a proper balance between rest and exercise to support recovery. Due to physical weakness after surgery, sufficient sleep is essential. Prolonged or excessive exercise should be avoided, and rest periods should be scheduled after physical activity to prevent fatigue-related setbacks in the rehabilitation process.	VR Timeline Simulation of Daily Rest and Exercise Planning: A VR simulation presents a full-day timeline illustrating the patient’s rest and exercise schedule. For example, the patient is shown going to bed at 10:00 p.m. and waking at 6:00 a.m., ensuring 8–10 h of restorative sleep. At 10:00 a.m., the patient performs 20 min of breathing exercises followed by 30 min of rest. At 3:00 p.m., a 15-min limb activity session is conducted, again followed by rest. Under this balanced regimen, physiological indicators remain stable, and recovery progresses steadily. In contrast, the simulation shows that insufficient rest or extended, uninterrupted exercise leads to excessive fatigue, with noticeable abnormalities in heart rate and blood pressure, and delayed recovery outcomes. This visual narrative underscores the critical importance of managing rest and exercise time appropriately to ensure effective rehabilitation.
	Monitoring Physiological Responses and Providing Timely Feedback: During pulmonary rehabilitation, patients must closely monitor their physical responses. If any abnormal symptoms arise—such as chest pain, dizziness, or shortness of breath—exercise should be immediately discontinued. Patients should promptly report these issues to healthcare providers to allow for appropriate evaluation and timely adjustment of the rehabilitation plan, ensuring both safety and efficacy.	VR Simulation Emphasizing Real-Time Monitoring and Response: The VR scenario depicts a pulmonary rehabilitation session with real-time monitoring panels displaying key physiological parameters, including heart rate, blood pressure, and oxygen saturation. At the outset of the exercise, all indicators are within normal ranges and the patient appears stable. However, as the session progresses, the patient suddenly experiences chest pain, accompanied by abnormal fluctuations in vital signs. The patient immediately halts the activity and alerts medical staff using a smart device or emergency call system. Medical personnel arrive promptly, conduct an assessment, and adjust the rehabilitation plan based on the patient’s condition. This simulated scenario highlights the critical importance of patient vigilance and timely feedback during rehabilitation to ensure a safe and effective recovery process.

**Table 2:** Pulmonary rehabilitation training content in the VR system.

Training content	VR presentation format
Pursed-lip breathing	In the VR scenario, the patient is guided to sit semi-recumbently and practice pursed-lip breathing. First, they are instructed to inhale slowly through the nose for approximately 2 s, then exhale gently through pursed lips (as if blowing a whistle) for 4–6 s. The training is synchronized with a human model and a visual lung animation. Each round lasts 4–6 s, repeated 10 times per session. Lung volume changes and airflow direction are dynamically shown.
Diaphragmatic breathing	In the diaphragmatic breathing module, the VR video guides the patient to sit or lie comfortably. A transparent overlay of the anatomical model displays abdominal expansion and retraction. The patient inhales for 3–4 s, allowing the abdomen to rise, then exhales slowly. This cycle is repeated 10–15 times. An embedded animation highlights diaphragmatic descent and pressure changes during inhalation. Real-time feedback and movement cues enhance technique accuracy.
Effective coughing	In the VR environment, the patient is first shown how to clear the airway. The system demonstrates deep inhalation for 2 s, holding the breath for 2 s, then segmented coughing (three short bursts) while bending slightly forward. The coughing motion is performed in sync with a visual animation of the respiratory tract. The sequence is repeated 2–3 times. The video emphasizes coordination between breathing and muscle contraction, highlighting points of force application.
Chest physiotherapy	The VR system displays standard chest physiotherapy techniques. The nurse is shown tapping the chest wall rhythmically (2–3 times per day), while the patient sits upright. The operator stands laterally, tapping with a cupped hand from top to bottom, along the lung segment. Frequency: 100–120 times/min, 3–5 min per location, total of 15–20 min. Lung segment diagrams assist with accurate positioning. Vibration and postural drainage techniques are also demonstrated.

(3) Video content development and system configuration: The development of VR video content was based on the scientific principles of postoperative pulmonary rehabilitation. Members of the research team collaboratively drafted the instructional scripts, ensuring the language was easy to understand, the procedural steps were clear, and the key points and challenges were emphasized. The script addressed rehabilitation needs at different postoperative stages. One senior nurse simulated the role of the patient, working with a bedside nurse to complete real-life filming. Video editing and post-production were completed with the technical support of the hospital’s information technology department. A total of six standardized 3D immersive instructional videos were produced, each with a duration of 3–5 min. The videos covered both education and training modules and supported personalized combination playback according to patient needs.

The videos adopted a 360-degree panoramic immersive format, incorporating multiple technologies such as step-by-step guidance, localized magnification, and interactive feedback to achieve visualization and interactivity of the training environment. During the instructional process, the system provided multidimensional demonstration content by combining animation with real-life footage. For instance, in the “pursed-lip breathing training” module, the main view displayed the nurse’s demonstration of breathing techniques, while a side-embedded animation window offered magnified and dynamic representations of key features such as lip positioning and airflow pathways. Simultaneously, a 3D lung model illustrated changes in lung volume and diaphragmatic motion during inhalation and exhalation, helping patients form a cognitive linkage between movement, physiological response, and accurate feedback through the “learn and do” approach. Similarly, in the “diaphragmatic breathing training” module, the video guided patients to recognize visible signals of abdominal elevation and retraction. A superimposed transparent anatomical animation dynamically displayed changes in thoracoabdominal pressure, alveolar expansion, and diaphragmatic movement throughout the respiratory cycle, thereby enhancing the patient’s understanding of the underlying physiological mechanisms. All videos supported functions such as pause, replay, and free-view switching. Patients could control and repeat the learning process using handheld controllers. Videos were uniformly formatted and imported into a head-mounted VR display system to ensure consistent playback quality and seamless information delivery across different devices.

The VR educational system was equipped with a head-mounted immersive display device, wireless interactive controllers, stereo headphones, a portable privacy screen, and a remote-control terminal, ensuring user comfort and privacy during training sessions. All equipment was accompanied by operation instruction cards and disinfection log sheets. Designated personnel were responsible for daily calibration, disinfection, and maintenance to ensure hygiene and the stable technical performance of the system under high-frequency usage conditions.

(4) Educational Process and Clinical Implementation: VR-based pulmonary rehabilitation education was officially initiated within 24 h after surgery. The responsible nurse assessed the patient’s vital sign stability, cognitive status, and ability to engage in autonomous training. Patients who met the criteria began viewing the VR educational content accordingly.

During the educational session, a nurse was required to remain present to monitor the patient’s engagement and physical responses, with particular attention to any adverse experiences such as dizziness, nausea, or anxiety. The nurse was also prepared to interrupt or adjust the session if necessary. Upon completion of the VR content, the nurse was responsible for documenting the following information in the “VR Education Implementation Record Sheet”: the date of the session, phase of rehabilitation, duration of the session, patient response (whether the VR session was completed and whether any discomfort occurred), nurse’s observations, and personalized notes (such as reasons for interruption or recommendations to postpone further training).

The VR education records were collected daily by the on-duty nurses and submitted to the head nurse for review. These records served as the basis for subsequent implementation of pulmonary rehabilitation exercises. If a patient experienced adverse reactions or demonstrated poor cooperation during the initial VR session, further VR training was suspended. A senior nurse then conducted a case-by-case reassessment to determine whether the patient should transition to conventional education and rehabilitation protocols. To ensure consistency and quality control in the educational process, all participating nurses received standardized training and strictly adhered to the operating guidelines. Upon completion of the VR education, each patient’s individual record was integrated into the nursing information system to align with subsequent rehabilitation implementation.

### 2.4 Observation indicators

① Pulmonary function parameters: On Day 0 (pre-intervention) and Day 3 post-intervention, pulmonary function was assessed using a spirometer. Key indicators included forced expiratory volume in 1 s (FEV_1_), forced vital capacity (FVC), and maximal voluntary ventilation (MVV); ② Incidence of postoperative pulmonary complications: Within 7 days after surgery, the occurrence of complications such as atelectasis and pulmonary infection was recorded, based on imaging findings and clinical diagnosis; ③ Satisfaction with health education: Before discharge, patients completed a nurse-administered questionnaire entitled “Pulmonary Rehabilitation Health Education Satisfaction Survey.” The scale included 10 items evaluating the format, content, practicality, clarity, and overall effectiveness of the educational intervention. A binary scoring system was used for each item (1 point for “satisfied,” 0 points for “dissatisfied”), with a total score of 5 points. A score ≥4 was defined as “satisfactory.” Satisfaction rates were calculated and compared between groups; ④ Safety monitoring: During the intervention, nurses recorded adverse reactions occurring while patients viewed the VR content. These included visual or auditory fatigue, reduced attention, dizziness, nausea, and disorientation. Any discomfort led to immediate discontinuation of the intervention and was reported to the research team to ensure safety. The number and incidence rate of adverse events in the intervention group were ultimately calculated as part of the safety assessment.

### 2.5 Statistical analysis

Statistical analysis was performed using SPSS version 26.0. Continuous variables were expressed as mean ± standard deviation. For data conforming to a normal distribution, between-group comparisons were conducted using the independent samples *t*-test. For non-normally distributed data, the Mann–Whitney U test was applied. Categorical variables were presented as frequency and percentage n(%), and group comparisons were performed using the chi-square (χ^2^) test. A *p*-value of <0.05 was considered statistically significant.

## 3 Results

### 3.1 Comparison of adherence rates and baseline characteristics between groups

A total of 95 patients were enrolled in both the intervention group and the control group. In the intervention group, two patients failed to complete the 3-day pulmonary rehabilitation program due to clinical deterioration, and three patients discontinued participation due to subjective factors such as fatigue or reluctance. In the control group, one patient withdrew due to clinical deterioration, while 13 patients dropped out due to fatigue or resistance. The pulmonary rehabilitation adherence rate was 94.74% in the intervention group and 85.26% in the control group. The difference was statistically significant (*χ*^2^ = 4.737, *P* < 0.05). Among patients who completed the pulmonary rehabilitation program, there were no statistically significant differences between the two groups in terms of baseline characteristics such as age, sex, and educational level (*P*>0.05), indicating comparability. Detailed data are presented in Table 3.

**Table 3:** Comparison of baseline characteristics between the two groups.

Variable	Intervention group (*n* = 90)	Control group (*n* = 81)	*t/χ* ^2^	*P*
Age (years)	58.16 ± 12.71	59.05 ± 11.17	0.510	0.611
Gender [n (%)]			0.010	0.920
Male	55(58.5)	52(57.8)		
Female	39(41.5)	38(42.2)		
Education level [n (%)]			2.020	0.568
Bachelor’s degree or above	10(10.6)	13(14.4)		
Associate degree	18(19.1)	21(23.3)		
High school	19(20.2)	20(22.2)		
Middle school or below	36(50.0)	47(40.0)		
Body weight (kg)	65.21 ± 9.91	65.62 ± 10.38	0.384	0.702
Smoking history [n (%)]			0.414	0.520
Yes	52(57.8)	46(56.8)		
No	38(42.2)	35(43.2)		
Operation time (h)	2.84 ± 1.42	2.64 ± 1.51	0.899	0.370

### 3.2 Comparison of pulmonary function parameters

Between the Two Groups Before the intervention, there were no statistically significant differences between the two groups in terms of FVC, FVC%, FEV_1_, FEV_1_%, MVV, and MVV%(*P*>0.05), indicating baseline comparability. After the intervention, the VR group showed significantly greater improvement in four standardized pulmonary function indicators—FVC%, FEV_1_%, MVV and MVV%—compared with the control group (*P* < 0.01 for all). However, no significant differences were observed between the groups in the absolute values of FVC and FEV_1_ (*P* > 0.05). Detailed results are presented in Table 4.

**Table 4:** Comparison of pulmonary function parameters between the two groups.

Group	FVC (L)		FVC (%)		FEV_1_ (L)		FEV_1_ (%)		MVV (L)		MVV (%)	
	Pre	Post	Pre	Post	Pre	Post	Pre	Post	Pre	Post	Pre	Post
Intervention group (*n* = 90)	2.74 ± 0.85	2.87 ± 0.62	57.98 ± 1.33	72.26 ± 1.70	2.33 ± 0.62	2.23 ± 0.71	56.36 ± 1.68	74.76 ± 1.92	48.50 ± 7.88	55.61 ± 7.55	69.03 ± 11.70	78.01 ± 12.56
Control group (*n* = 81)	2.75 ± 0.78	3.01 ± 0.60	57.95 ± 1.34	65.95 ± 1.78	2.21 ± 0.65	2.28 ± 0.64	56.74 ± 2.06	66.45 ± 2.09	47.66 ± 7.08	47.55 ± 7.48	69.35 ± 9.85	72.19 ± 10.01
*t/Z*	−0.013	0.434	0.148	23.694	1.147	−0.492	−1.306	27.061	0.734	7.002	−0.196	3.327
*P*	0.99	0.144	0.883	<0.01	0.253	0.623	0.193	<0.01	0.462	<0.01	0.845	<0.01

### 3.3 Comparison of extubation time and postoperative complications between the two groups

More than 80% of patients in both groups underwent extubation within the first 3 days after surgery. Statistical analysis showed that the VR group had a significantly shorter extubation time compared to the control group (*p* < 0.01). Regarding postoperative complications within 7 days after surgery, the incidence of hospital-acquired pneumonia was 6.38% in the VR group and 6.67% in the control group. The incidence of atelectasis was 8.51% in the VR group and 6.67% in the control group. No statistically significant differences were observed between the groups for either complication (*P* > 0.05). Detailed results are presented in Table 5.

**Table 5:** Comparison of chest tube removal time and postoperative complications between the two groups.

Group	Chest tube removal time (h)	HAP (n, %)	Atelectasis (n, %)
Intervention group (*n* = 90)	84.71 ± 17.05	6(6.7)	8(7.4)
Control group (*n* = 81)	96.10 ± 18.01	6(5.7)	6(6.6)
*t*/*χ*^2^	−4.256	0.036	0.124
*P*	<0.01	0.850	0.724

### 3.4 Comparison of patient satisfaction between the two groups

A total of 82 patients (91.11%) in the intervention group reported satisfaction, compared with 59 patients (72.84%) in the control group. The difference between the two groups was statistically significant (*χ*^2^ = 14.548, *p* < 0.01).

### 3.5 Adverse reactions in the intervention group

During VR sessions, two patients in the intervention group experienced dizziness and one patient reported fatigue. These symptoms improved promptly after the sessions were stopped, and no recurrence was observed upon subsequent use.

## 4 Discussion

In the early postoperative stage of lung cancer patients, the recovery of pulmonary ventilation function is of significant clinical importance for enhancing overall rehabilitation quality, improving spontaneous breathing, and facilitating early readiness for extubation (7). Although active pulmonary function exercises—such as pursed-lip breathing, diaphragmatic breathing, and effective coughing—have been widely incorporated into postoperative pulmonary rehabilitation protocols, many patients still experience limitations in training outcomes and poor execution quality due to postoperative fatigue, anxiety, insufficient cognitive understanding, or incorrect mastery of technical skills (8).

This study introduced a virtual reality-based health education system precisely to address the key barrier of “behavioral implementation failure” in postoperative pulmonary rehabilitation. By providing an immersive, multisensory interactive experience, VR transforms abstract respiratory training movements into visualized, stepwise procedures. This approach helps patients quickly understand the principles of breathing exercises, correct improper techniques, and enhance memory retention and skill acquisition through repeated viewing and practice. As a result, the VR intervention improves training quality within the cognitive–execution–feedback cycle and promotes effective recovery of pulmonary function during the early postoperative stage (9).

### 4.1 Effect of virtual reality-based health education combined with pulmonary function training on early postoperative pulmonary recovery

The study results showed that, by postoperative day 3, patients in the intervention group demonstrated improvements in MVV, MVV%, FEV_1_% and FVC% compared to their levels on day 1. Moreover, the magnitude of improvement was significantly greater than that observed in the control group. These findings suggest that a VR-assisted pulmonary rehabilitation intervention can effectively promote early recovery of pulmonary function following surgery.

In this study, maximal voluntary ventilation (MVV), an important parameter reflecting the overall coordination and endurance of the respiratory muscles, showed significantly greater improvement in the intervention group by postoperative day 3 compared to the control group. This suggests that VR-guided postoperative pulmonary training may enhance patients’ ventilatory reserve capacity. Quist et al. (10) reported that MVV significantly improves following postoperative rehabilitation in thoracic cancer patients, making it a sensitive indicator of early respiratory recovery. Similarly, Zhang et al. found that graded exercise rehabilitation based on pulmonary function classification significantly improved MVV and exercise capacity in elderly lung cancer patients, with greater improvements observed over time (11). In the present study, the application of VR technology enhanced visual feedback and rhythm guidance during training, allowing patients to perform breathing exercises more accurately under nursing supervision. This approach likely increased respiratory muscle engagement and training intensity, thereby contributing to the improvement in MVV levels.

The intervention group showed significantly greater improvement in FEV_1_% and FVC% compared to the control group, indicating that even after partial resection of lung tissue, VR-guided pulmonary function training can enhance alveolar ventilation coordination and airflow dynamics. Duan et al. (8) reported that postoperative respiratory training in lung cancer patients undergoing lobectomy or sublobar resection significantly improved FEV_1_ and FVC in a subset of patients, with particularly pronounced effects observed in those who underwent sublobar resections. The study also pointed out that traditional one-way education delivered by nursing staff may temporarily improve patient compliance, but the lack of personalized interaction and real-time feedback often leads to poor adherence to training and limited overall pulmonary function recovery. Moreover, in patients undergoing lobectomy, although respiratory training did not directly enhance pulmonary function parameters, structured educational interventions showed a positive effect in alleviating postoperative anxiety, suggesting a potential synergistic relationship between psychological support and training outcomes. In the present study, the VR-based educational model offered mechanisms of active immersion and contextual interaction, enabling patients to engage in mimetic exercises while watching procedural demonstrations. This approach enhanced the accuracy and continuity of exercise performance, thereby contributing to improvements in FEV_1_% and FVC% at the functional level (6).

It is noteworthy that in this study, both groups exhibited an upward trend in the absolute values of FEV_1_ and FVC by postoperative day 3, although the intergroup differences did not reach statistical significance. This may be attributed to the fact that all enrolled patients underwent lobectomy, where the irreversible resection of lung tissue leads to a significant baseline reduction in lung volume. As a result, the short-term recovery of absolute values is structurally constrained. Zou et al. (12) reported that during the early postoperative period, improvements in FVC and FEV_1_ were limited, indicating a delayed recovery pattern for absolute pulmonary function. The findings of the present study are consistent with this, suggesting that while respiratory training may enhance breathing efficiency, its short-term effect on increasing absolute lung volume remains restricted by the extent of intraoperative tissue resection and the recovery rhythm of the postoperative thoracic condition.

### 4.2 Effects of virtual reality-based health education on chest tube removal time and pulmonary infections

The results of this study indicated that the postoperative chest tube removal time was significantly shorter in the intervention group compared to the control group, suggesting that virtual reality-based health education combined with pulmonary function training may help accelerate the termination of thoracic drainage. However, there were no statistically significant differences between the two groups in the incidence of hospital-acquired pneumonia (HAP) and atelectasis within 7 days postoperatively. This finding implies that although the intervention played a positive role in promoting pulmonary function recovery, its short-term impact on preventing pulmonary complications remains limited.

The duration of postoperative chest tube placement is closely related to the degree of lung re-expansion, pleural drainage volume, and gas exchange efficiency. Previous studies have shown that lung cancer patients who achieve adequate early lung inflation and effective sputum expectoration are more likely to meet the criteria for chest tube removal at an earlier stage (13). Zhang et al. (14) reported in a comparative study on chest tube removal timing among thoracic surgery patients that those who received targeted respiratory training experienced a more rapid decline in drainage volume and more complete lung re-expansion by postoperative days 2–3, with an average chest tube removal time shortened by approximately 1–1.5 days compared to those receiving routine care. In this study, patients in the intervention group who received VR-based health education were able to acquire techniques such as deep inhalation, breath-holding, and effective coughing at an earlier stage. The training process was more standardized and repeatable, which helped enhance diaphragmatic excursion and alveolar tension, thereby physiologically promoting lung re-expansion and pleural fluid absorption. Additionally, the improvement in pulmonary ventilation brought about by respiratory training may also reduce intrathoracic negative pressure fluctuations caused by ineffective ventilation, contributing to a shorter duration of chest tube placement. A study by Ding et al. (15) also observed that patients who underwent early pulmonary function training had shorter chest tube removal times and faster recovery of lung tension. Although VR technology was not incorporated in that study, the findings support the observations of the present study—namely, that enhancing exercise adherence and execution quality can promote thoracic functional recovery.

However, despite the significant improvements in pulmonary function indicators and earlier chest tube removal observed in the intervention group, no statistically significant differences were found between the two groups in the incidence of atelectasis and hospital-acquired pneumonia (HAP) within 7 days postoperatively. This suggests that while pulmonary function improvement provides a foundation for preventing pulmonary complications, it is not sufficient on its own to determine their occurrence. The pathogenesis of HAP and atelectasis is multifactorial and complex, involving not only lung re-expansion status but also factors such as the patient’s immune function, sputum characteristics, positioning management, and use of sedative medications. Liang et al. (16) indicated that respiratory training has certain preventive value against pulmonary complications, but its effectiveness is more evident in patients with high-risk factors or impaired consciousness. Moreover, it requires integration with multimodal nursing interventions—such as suctioning, postural drainage, and nebulization therapy—to establish a comprehensive and effective management cycle. In the present study, all included patients were post-operative, conscious, and able to cooperate autonomously with training, resulting in a relatively low baseline risk of infection and an overall low incidence of atelectasis and HAP. Under these circumstances, although the intervention group demonstrated superior pulmonary function recovery, the intergroup differences in complication rates may have been difficult to detect. Furthermore, studies have shown that HAP and atelectasis commonly occur within 5–10 days after surgery (17). Since this study limited postoperative observation to 7 days, the medium- to long-term effectiveness of the intervention on controlling pulmonary infections and atelectasis warrants further follow-up.

In summary, this study preliminarily demonstrates that a health education model based on virtual reality (VR) technology can improve the early postoperative pulmonary rehabilitation process in patients with lung cancer. In particular, it shows advantages in enhancing standardized pulmonary function indicators such as MVV, FEV_1_%, and FVC%, and in shortening the duration of postoperative chest tube placement. However, several limitations should be acknowledged. First, the study was designed as a single-center, non-concurrent controlled trial with a relatively small sample size, which may limit the generalizability and representativeness of the findings. Second, the observation period covered only postoperative days 1 through 3, which is insufficient to evaluate the sustained effects of the intervention on pulmonary complications, long-term pulmonary function, or overall hospitalization outcomes. Future studies should expand the sample size, optimize study design, and extend the follow-up period to systematically assess the long-term value and application boundaries of VR technology in postoperative pulmonary rehabilitation management, thereby providing stronger clinical evidence for its widespread use in nursing practice.

## Data Availability

The raw data supporting the conclusions of this article will be made available by the authors, without undue reservation.
